# Developmental Trajectory of Depressive Symptoms in Chinese College Students: Latent Classes and Gender Effect

**DOI:** 10.3390/ijerph19063508

**Published:** 2022-03-16

**Authors:** Shegang Zhou, Lin Jin, Xiaoxian Liu, Xiaosheng Ding, Xiangru Zhu

**Affiliations:** 1 Department of Psychology, Institute of Education, Henan Normal University, Xinxiang 453007, China; 463009@htu.edu.cn (S.Z.); 111028@htu.edu.cn (X.L.); 041079@htu.edu.cn (X.D.); 2 Mental Health Center, Henan Normal University, Xinxiang 453007, China; 463015@htu.edu.cn; 3 Institute of Cognition, Brain and Health, Henan University, Kaifeng 475004, China

**Keywords:** depressive symptoms, trajectories, college students, gender differences

## Abstract

Depressive symptoms are prevalent in Chinese college students, but little is known about the heterogeneity in the developmental trajectory of depressive symptoms in China. This study examined heterogeneity in the development of depressive symptoms and examined the effect of gender on the developmental trajectories over a 14-month period among Chinese college students (N = 1163, mean age 20.18, 80.31% female). Three different trajectories, moderate-increasing, high-stable and low-stable, captured the heterogeneity in the development of depressive symptoms. Gender showed significant influence on class membership. Relative to the moderate-increasing class, males emerged as significantly more likely than females to be found in the low-stable class (odds ratio (OR) = 2.73, 95% CI = (1.21, 6.13), *p* = 0.015) and the high-stable class (OR = 5.10, 95% CI = (1.12, 23.18), *p* = 0.035). The results provide additional evidence for the conclusion that the trajectories of depressive symptoms are heterogeneous with Chinese samples. Moreover, cultural difference should be paid more attention to when examining the effect of gender and other predictors of the trajectories of depressive symptoms.

## 1. Introduction

Depressive disorder is a highly prevalent and impairing condition which affects people of all ages worldwide [1]. At a global level, over 300 million people are estimated to suffer from depression, equivalent to 4.4% of the world’s population [2]. In China, the first nationally representative survey on mental disorders showed that mood disorders were the second most prevalent class of lifetime disorders (7.4%), and major depressive disorders was the most prevalent mood disorder (lifetime prevalence 3.4% and 12-month prevalence 2.1%) [3]. The prevalence of depressive symptoms is usually several times that of depressive disorder. One of the major groups especially vulnerable to depressive symptom is college students [4]. According to the previous meta-analyses, the prevalence of depressive symptoms is higher in college students than non-college students or the general population [5,6]. Many previous studies reveal that college students vary strongly from each other in how their depressive symptoms change [7]. Gender is related to the development of depressive symptoms [8]. The aim of the present study was to explore how the gender factor influences the developmental trajectory of depressive symptoms among Chinese college students.

A systematic review of worldwide studies of depressive symptom prevalence among college students between 1990 and 2010 showed that the prevalence rates ranged from 10% to 85% with a weighted mean prevalence of 30.6% [9]. In China, a recent meta-analysis indicated that the prevalence of depressive symptoms among Chinese college students is 23.8% (95% CI: 19.9–28.5%) [6]. The majority of college students remain low in depressive symptoms, but some college students experience an increase in level of depressive symptoms [8]. Individuals who experience an increase in depressive symptoms or persistence of high level in depressive symptoms are at high risk for poor social and psychiatric outcomes [10,11]. Therefore, it is important to identify factors that predict which college students are most likely to experience such stable high or enhanced depressive symptom trajectories.

Notably, gender disparity in the prevalence of depression is extensively reported. From a global perspective, there is generally reported to be twice as many females experiencing major depressive disorder as males [12]. In China, the gender difference in the prevalence of depressive disorder is mixed. For the adult population, there is significant gender difference in weighted 12-month prevalence of major depressive disorder (female 2.5% vs. male 1.7%). However, there is no significant gender difference in weighted 12-month prevalence of dysthymic disorder, which is characterized by chronic mild depression (female 1.1% vs. male 1.0%). The gender difference of weighted 12-month prevalence of depressive disorder not otherwise specified was marginally significant (female 1.6% vs. male 1.2%, *p* = 0.056) [3]. For college students, an important study (N = 5245) combining a questionnaire and interviews indicated the prevalence of major depressive disorder was 4.0% of Chinese university students [13]. However, because about a quarter of the high-score students refused to participate in the interview, the authors did not report whether there was a significant gender difference.

In Western cultures, the existence of a gender difference in depressive symptoms is also well established. Gender differences in depressive symptoms have been identified by many studies. In English-speaking countries, prevalence rates of depressive symptoms among women are generally higher than among men [14]. However, the findings have been different in China. A meta-analysis that estimated the prevalence of depression among adolescents in China showed that the prevalence of boys (16.80%) was higher than the girls (15.60%) [15]. Another meta-analysis among Chinese college students over the past 10 years reached the same conclusions, in which the prevalence of boys was 33.27% and of girls was 32.94% [16]. A study among freshmen also found the prevalence of depression symptoms was significantly higher among male students (27.5%) than among female students (21.9%) [17]. The reason for this may be that male freshmen feel more social pressure than female freshmen in China [16]. Moreover, when experiencing mental health issues, female students generally hold more active help-seeking attitudes than males [18]. Given the differences in the gender effect on depressive symptoms among college students between English-speaking countries and China, it is worth evaluating whether there are differences in the effect of gender on developmental trajectories too.

The identification of distinct trajectories of depressive symptoms is a major topic of study in the past 15 years. One of the methods, called growth mixture modeling (GMM), offers the opportunity to estimate distinct groups of individual trajectories. GMM assumes the developmental trajectories of an outcome measure may be heterogeneity. The entire sample could be separated into more homogeneous subgroups, referred to as “class”, based on close similarities in developmental trajectories [19]. Trajectory-based studies are important for classifying etiologically distinct subgroups and the recovery potential of individuals with elevated symptoms early in life [19]. The identification of distinct trajectories of depressive symptoms can help to detect different pathways of development, search for possible different causes for different developmental groups, and implement adequate preventive strategies [20].

Evidence suggested that the symptom trajectories of depression are heterogeneous. A systematic review of studies that examined heterogeneity in long-term (5 years+) trajectories of depressive symptoms showed that either three or four distinct trajectory classes have been consistently documented. Trajectories varied in terms of severity (low, medium, high) and stability (stable, increasing, decreasing), for instance, stable low class, persistently high class, stable moderate class, increasing class and decreasing class. In most studies, the majority of participants had consistently few or no depressive symptoms, but a notable minority (usually <10%) reported persistent symptoms [21]. Another review of studies among children and adolescents indicated that between 3 and 11 trajectory classes were identified. A random pooled effect estimate identified 56% (95% confidence interval (CI): 46–65%) of the populations on a “no or low” trajectory (stable low, minimal or mild depression symptoms) and 26% (CI: 14–40%) on a “moderate” trajectory (persistently moderate depressive symptoms). “High” (persistently high depressive symptoms), “increasing” (depressive symptoms are increasing over time), and “decreasing” (depressive symptoms are decreasing over time) subgroups were evident for 12% (CI: 8–17%) [22]. Predictors of membership in a trajectory class included female gender, lower income/education/socioeconomic status, non-white race, higher stress reactivity, conduct issues, substance misuse, and problems in peer and parental relationships [21].

Some studies examined the effect of gender on the patterns and class membership of depressive symptom trajectories. However, the research conclusions are inconsistent. Some studies found similar trajectory patterns for boys and girls, but female gender was associated with the high symptoms class [23,24,25]. Castelao and Kroner-Herwig [20] found different trajectory class number between genders, with four classes for girls and three for boys. Girls also showed higher levels than boys and an increase of depressive symptoms over time double that of boys. Three studies examined the heterogeneity and the gender differences in developmental trajectories of depressive symptoms in Chinese college students. Li et al. [26] identified four trajectory classes over a one-year period: decreasing, low, high and increasing. A larger percentage of the high and the decreasing classes were girls, whereas more of the increasing depressive symptom class was boys. Liu and Liu [27] identified three trajectory classes over a three-year period, group relief, group risk and group deterioration, but they did not discover significant gender effect on the trajectories. Song et al. [8] found that for the no depressive symptom respondents at baseline, female college students were less likely to manifest depressive symptom in the following two years.

Liu et al. [27] conducted surveys once a year and conducted a total of three surveys in three years. The time interval between surveys was as long as one year, which is likely to obscure the developmental trajectory of depressive symptoms. Li et al. [26] only recruited 340 college students, and the small sample size can compromise the conclusions. Song et al. [8] only explored the incidence of depressive symptoms among the no depressive symptom respondents at baseline. The purposes of the present study were to identify distinct developmental trajectories of depressive symptoms over a 14-month period among Chinese college students and to examine whether gender has an effect on those trajectories. Our research plans to recruit more college students and use shorter-interval surveys. Based on prior research, we predicted that gender can predict the trajectory of depressive symptoms.

## 2. Materials and Methods

### 2.1. Participants and Procedure

To obtain a representative sample, a cluster random sampling method was employed to select the study participants from the freshmen of Henan Normal University. First, we recruited the freshmen from all the majors in the first semester of the academic year 2019–2020. The students in a same major were formed into a cluster. Then, each cluster was numbered, and 20 clusters were randomly selected using a random-number table. All the students in the selected clusters were enrolled in this study. The survey was conducted one month after the freshmen entered the college. After arranging a survey date with the student advisors, interviewers visited each cluster and explained the purpose and procedures of the study to the samples to encourage them to participate. Written informed consents were obtained from all participants. Then, all participants completed the baseline assessment which was set up on a professional online survey platform named Wen Juan Xing using their mobile phone. The baseline assessment was conducted in October in 2019 (T1), and the follow-up assessments took place at four time points: T2 (February 2020), T3 (May 2020), T4 (August 2020), T5 (November 2020). The Ethics Committee of the Institute of Education, Henan Normal University, approved the protocol of this study (project identification code 2019BJY012).

A total of 1177 students within the 20 clusters were selected for the baseline assessment. Of those, 14 did not complete follow-up assessments at any time point because of suspension from school and any other reasons. The final analytic sample was 1163. Participants were mostly female (80.31%) and ranged in age from 18 to 24 years (M = 20.18, SD = 0.89) at baseline assessment. None of the students received any compensation to complete the study.

### 2.2. Measures

Zung Self-Rating Depression Scale (SDS) is a widely used instrument to measure depression [28]. The instrument is composed of 20 items with a four-point scale (1 = not true at all, 4 = true all the time). The raw score was multiplied by 1.25 to obtain a standard score. Standard scores for evaluation of the severity of depression are as follows: normal score with upper limit is 52, mild depression is 53–62, severe depression is 63–72 and extremely severe depression is higher than 73 [28,29]. Cronbach’s alpha coefficient of this scale was over 0.79 for each of the 5 measurement times.

### 2.3. Data Analysis

GMM was used to identify classes of depressive symptom trajectories using the statistical software program Mplus, Version 8.0. Unlike traditional Latent Growth Models where a single trajectory is estimated, through GMM it is possible to identify different classes of trajectories, that is, trajectories that may vary in the form of the growth or change over time [30,31,32,33]. Starting with a single-class growth curve model, models with different numbers of trajectories (2 to 4) were estimated. Then, the optimal number of latent classes were selected according to criteria suggested in the literature [30,31,32,33], including Akaike’s Information Criterion (AIC), Bayesian Information Criterion (BIC), sample-size adjusted BIC (ABIC), entropy value, Vuong–Lo–Mendell–Rubin likelihood ratio test (VLRT) and bootstrapping likelihood ratio test (BLRT). Lower AIC, BIC and ABIC values are indicative of better model fit. Both VLRT and BLRT test a model with K classes versus a model with K−1 classes. A significant *p*-value indicates that the model with K classes is better than the model with K−1 classes, and a non-significant *p*-value indicates that the model with K classes is not an improvement over the model with K−1 classes. The classification accuracy can be considered through the entropy value (higher than 0.7). In addition to these criteria, there are also requirements for class size, that is, no class should have fewer than 5% of the sample in the model. Once the model was identified, the effect of gender on the trajectories was examined by conditional models and multinomial logistic regression. All models were estimated with 500 random sets of start values to find the true maximum likelihood solution and avoid local solutions.

## 3. Results

### 3.1. Descriptive Statistics

Table 1 presents the demographic characteristics and depression groups of the participants at baseline assessment. At the baseline assessment, the proportions of normal scores, mild depression, severe depression and the extremely severe depression were 60.6%, 34.9%, 4.5% and 0%, respectively. Table 2 presents the means and standard deviations for depressive symptoms and correlations between depressive symptoms at every measurement time. There was a significant correlation between the depression symptoms at each time. Independent sample t-tests demonstrated that there were no significant differences (between *p* = 0.197 and *p* = 0.855) on depressive symptoms between girls and boys at all five assessment points. Repeated-measure one-way ANOVA indicated that the samples showed low to moderate levels of depressive symptoms with a significant increasing trend over the period (F = 441,074, *p* < 0.001). The depressive symptoms at first assessment point were the lowest (*p* < 0.001) among the five assessment points, and the score at the third assessment was the highest (*p* < 0.001).

### 3.2. Trajectory Model

Consistent with recommendations for model testing, the analysis started with a basic linear growth model in which the entire sample was considered as belonging to a single homogenous group. Then, two- to four-class GMMs were conducted to ascertain the optimum GMM model. The relative fit indices for all models are presented in Table 3. For all the models, the BLRT values are significant. The VLRT showed that the three-class solution fit significantly better than the two-class solution, but the four-class solution did not fit significantly better than the three-class solution. This suggested that the three-class model could be considered optimum. The AIC, BIC and ABIC values decreased steadily from the two- to three-class models, which indicated preference for the three-class solution. However, the entropy value for the two-class solution was higher than the three-class solution, indicating that the two-class model is better. Research suggested that BIC should weight more strongly in comparisons within model sets, because it performed best of the information criteria [31]. Based on this consideration, the three-class model was selected.

**Figure 1:**
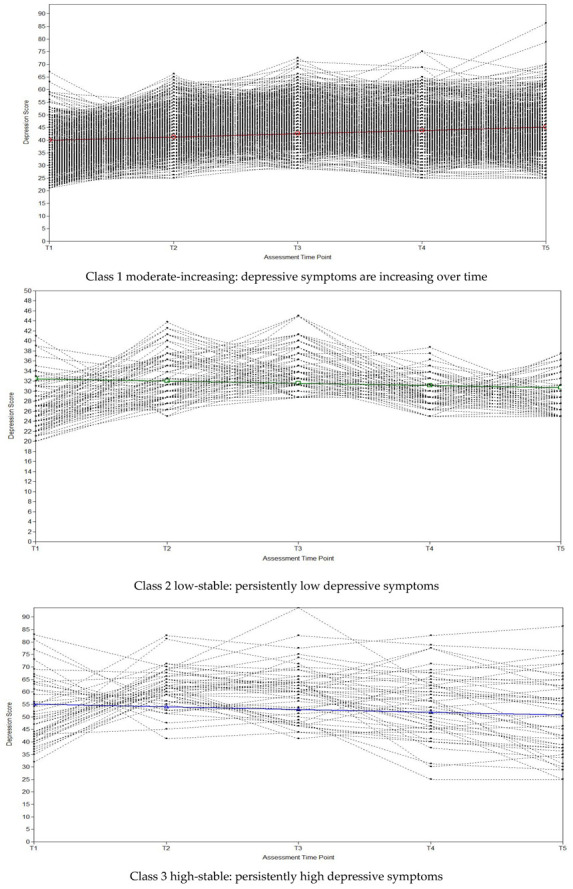
Estimated means and observed individual growth trajectories for each class.

The estimated means and observed individual values of the three-class solution are shown in Figure 1. The first class, containing the majority of students (83.73%), was labeled moderate-increasing, which demonstrated a significant upward trend of depressive symptoms, with a significant mean intercept (M = 39.98, SE = 0.61, *p* < 0.001) and a significant mean slope (M = 1.29, SE = 0.15, *p* < 0.001). The second class, consisting of 8.69% of the sample, was labeled low-stable which demonstrated a stable course, with a significant low intercept (M = 32.50, SE = 0.99, *p* < 0.001) and a non-significant decreasing slope (M = −0.45, SE = 0.26, *p* = 0.078). The third class, consisting of 7.58% of the sample, was labeled high-stable, which started with a significant high mean intercept (M = 55.11, SE = 2.89, *p* < 0.001), but showed a non-significant decreasing slope (M = −1.10, SE = 0.91, *p* = 0.227). The identification of the three different trajectories supported the hypothesis that there were also different classes of trajectories of depressive symptoms in Chinese college students.

### 3.3. Gender as a Predictor of Trajectories

Conditional models with gender as the independent variable and multinomial logistic regression were conducted to examine whether gender predicted trajectory growth factors and class membership. Based on the relative fit indices, the three-class solution was chosen in the conditional model again. In the three-class conditional model, the AIC, BIC and ABIC were 36,473.782, 36,572.547 and 36,509.025, respectively. The decrements of these indices indicated that inclusion of gender as an independent variable significantly improved model fit over the unconditional model. The entropy value increased from 0.768 to 0.878, indicating better classification accuracy. This result was also supported by the VLRT (*p* = 0.007) and BLRT (*p* = 0.008). The conditional model showed that the inclusion of gender as a predictor did not change the unconditional model in a substantial way with regard to classification shape and characteristics of each class.

However, gender showed significant influence on class membership. The multinomial logistic regression analysis indicated that relative to the moderate-increasing class, males emerged as significantly more likely than females to be found in the low-stable class (odds ratio (OR) = 2.73, 95% CI = (1.21, 6.13), *p* = 0.015) and the high-stable class (OR = 5.10, 95% CI = (1.12, 23.18), *p* = 0.035).

## 4. Discussion

The aim of this study was to identify different classes of developmental trajectories of depressive symptoms in Chinese college students and to examine whether gender had influence on the trajectories. Three different classes of trajectories, moderate-increasing, low-stable and high-stable, were identified which captured the heterogeneity in the development of symptoms during the 14-month follow-up period.

The finding that the developmental trajectories of depression symptoms are heterogeneous is consistent with those of previous longitudinal studies with longer time intervals between each assessment. It supports the view that heterogeneity in depression trajectories exists across cultures. Moreover, it indicates that even during a shorter period, there are subgroups of Chinese college students who follow unique trajectories of depressive symptoms.

In this analysis, three classes of trajectories were identified. In previous studies, the number of trajectory classes ranged from 3 to 11, with most of the studies identifying 3 or 4 classes [21,22]. The stable-low class and persistently high class were the two trajectory patterns observed frequently in earlier studies. Consistent with the earlier studies, our study also identified these two classes. The third trajectory pattern identified in our study is moderate-increasing class, which was also identified in some studies among adults and children. Chaiton et al. [25] identified a moderate trajectory group with linear increase among adolescents during grade 7 to grade 11. Costello et al. [34] found a late escalating group among participants from age 12 to 25, whose level of depressed mood resembled that of the no depressed mood group in early adolescence but climbed steadily into young adulthood. Both of the two studies in Chinese college students identified an increasing depression class [26,27].

In addition to the increasing trajectory, Li et al. [26] and Liu et al. [27] identified a decreasing class whose depression level declined notably with time. Costello et al. [34] also found an early high declining depressed mood group which was characterized by relatively high depressed mood from early to mid-adolescence, followed by a steady decline over time. The decreasing trajectories were observed among child [20,23], adolescent [35,36] and adult samples [37,38]. However, the decreasing trajectories were not identified in our study.

Taking the sizes of identified trajectories into account, a minority of students (7.58%) belonged to the high-stable class in our study. This was consistent with earlier reports. The previous studies found that a small proportion (usually <10%) of individuals across different populations and age groups persistently reported high levels of depressive symptoms for extended periods of time [36,39,40,41,42]. However, unlike previous studies, most students (83.73%) in our study belonged to the moderate-increasing class and a minority of students (8.69%) belonged to the low-stable class. The previous studies found that the majority of participants had consistently low depressive symptoms. Brière, Janosz, Fallu and Morizot [35] found that the stable-low class included mostly adolescents (68.1%) from age 12 to 16 years. During a 14-year follow-up of Canadian youths aged 10 to 25 years, Ferro, Gorter and Boyle [43] found the largest class (55%) consisted of youths with low symptom levels and the subclinical trajectory class included 39% of youths.

Differences in the number and sizes of trajectories between our study and previous studies are likely attributable to differences in research objects and research methodologies. Our research objects are Chinese college students, but the objects of most previous studies were children, adolescents, and adults among English-speaking countries. Cultural differences may result in differences in the manifestation of depressive symptoms [44]. Costello, Swendsen, Rose and Dierker [34] found that Asian American students were both more likely to be in the “low depression” and “early high depression” classes relative to the “no depression” class. This difference in manifestation of depressive symptoms may explain why most students belonged to the moderate class in our study. Furthermore, measurement occasions and the follow-up period could affect the number and sizes of trajectories. Song et al. [45] observed the changes in depressive symptoms among Chinese college freshmen for two-year periods. They found the depressive scores significantly increased in the second and third surveys, then decreased in the fourth and fifth surveys. Our study provided more measurement occasions, and the follow-up period was shorter. This may reduce variability among trajectories, resulting in the identification of three classes.

Most notably, the results revealed a general upward trend of depressive symptoms during the students’ first 14 months at college. Cheng et al. [46] found that the depression symptom levels of Chinese college freshmen increased in a quadratic linear trajectory within four months after entering college. The results of the two studies seem to be in accord with each other. The upward trend of depressive symptoms may be related with the transition to college. After entering college, the students are exposed to variety of stressors, such as academic burden, the change of environment and relationship, worries about the future, the reduction of social support from teachers, the decline in subjective social status and so on [46,47,48]. These stressors may trigger or exacerbate the depression symptoms of the students [49].

This study found that gender showed significant influence on class membership. Relative to the moderate-increasing class, males emerged as significantly more likely than females to be found in the low-stable class and the high-stable class. Many studies confirmed the effects of gender in predicting membership to different trajectories of depressive symptoms. However, in these studies, the proportion of participants in classes with higher symptom burden was larger for females than for males [24,25,38,50,51]. One important reason may be different cultural backgrounds. In a study on the developmental trajectories of Chinese adolescent depressive symptoms, Hou and Chen [52] found that Chinese adolescents followed different patterns of depressive symptoms from U.S. youths. Specifically, girls followed an inverted U-shaped trajectory with a larger age range than U.S. girls, while boys’ depressive symptoms increased linearly with time. The results of our study are in line with the findings of Hou and Chen. Moreover, the risk factors associated with depressive symptoms are culture dependent. Greenberger, Chen, Tally and Qi [53] revealed that the quality of family relationships and grades in school had significantly stronger associations with depressive symptoms among Chinese youths than among U.S. youths. It suggested that cultural differences should be paid more attention when examining the effect of gender and other predictors on the trajectories of depressive symptoms.

In addition to cultural backgrounds, another reason may be gender differences in coping responses on life events. Life events and coping responses are significantly related to depression. Niu et al. [54] found that coping responses mediated the relationship between negative life events and depression and gender moderated the mediation role of coping responses among Chinese college students. When life events increased, male college students had more positive coping responses than female students; therefore, more female students had depressive symptoms and were found in the moderate-increasing class. However, when life events increased to a certain extent, male students not only had more positive coping responses but also more negative coping responses. Because of gender stereotypes [55], negative coping responses are considered to be a personality trait that men should not have. With the increase of negative coping responses, male students will blame themselves more for their performance. Their depressive experience will increase. Moreover, because of traditional gender roles in China, male college students are under greater pressure than female students. This collectively can explain why more male college students were found in the high-stable class.

The findings in the current study have some practice implications. Since the study revealed a general upward trend of depressive symptoms during the students’ first 14 months at college, colleges should pay more attention to prevent and intervene depression. Moreover, the students who followed different development trajectories of depressive symptoms may need different forms and amounts of mental health services. For the high-stable class, early detection and treatment seem particularly important since high depressive symptoms persist for a longer period in those students. For the moderate-increasing class, teaching them more techniques and strategies for regulating depression may be useful since their depressive symptoms did not reach the cut-off. In particular, the number of this class was particularly large and there were more female students than male students in this class. It is very important to involve more students in the prevention of depression, especially female students.

There are also some limitations to this study. First, the present sample was predominantly female and from one college in central China. Additional research is needed to replicate the findings of this study in college student samples with a higher representation of men and region. Second, the follow-up period with assessments was not long enough to capture the development trajectories of the students’ depressive symptoms throughout college. Future research should examine the results of this study during the whole college period, as this may allow for the emergency of additional classes as students have more opportunity to adapt to college over the course of their undergraduate careers. Furthermore, more research is needed to identify the development trajectories of depressive symptoms during the transition from adolescence to college student, which will be useful to understand the heterogeneity in the course of depressive symptoms. Third, besides gender, there are many variables that may influence the course of depressive symptoms, such as stressful life events, previous history of psychopathology, self-esteem and so on. More studies are needed to advance the understanding of factors that are associated to the mechanisms that account for college students moving in and out of particular trajectories.

## 5. Conclusions

Overall, this study provides additional evidence for the heterogeneity in the development trajectories of depressive symptoms from a different cultural context. Three subgroups, moderate-increasing, high-stable and low-stable, were identified in Chinese college students during the 14-month measurement period. The findings strongly recommend the development of more effective preventive and treatment interventions based on the different patterns of depressive symptoms. In particular, taking the moderate-increasing class into account, which accounted for the largest proportion, more attention must be paid to enhance mental health services for Chinese college students. Gender showed significant influence on class membership. However, contrary to the research in the Western cultural context, males emerged as significantly more likely than females to be found in the low-stable class and the high-stable class. It suggested that cultural difference may influence the effect of gender and other predictors on the trajectories of depressive symptoms.

## Figures and Tables

**Table 1:** Demographic characteristics of participants.

	N	%
Gender		
Male	229	19.69
Female	934	80.31
Parents’ marital status		
Married to each other	1039	89.34
Separated	19	1.63
One or both dead	30	2.58
Divorced	45	3.87
Did not marry each other	30	2.58
Mother’s education		
Junior school	272	23.39
Junior high school	502	43.16
High school	250	21.50
Some college/vocational school	72	6.19
4-year college	62	5.33
Master’s degree	5	0.43
Father’s education		
Junior school	172	14.79
Junior high school	496	42.66
High school	316	27.17
Some college/vocational school	97	8.34
4-year college	81	6.96
Master’s degree	1	0.08
Household income (monthly income per capita)		
Low (CNY < 2000)	626	53.82
Medium (CNY 2000–5000)	444	38.18
High (CNY > 5000)	93	8.00
Depression group		
Normal	705	60.62
Moderate	406	34.91
Severe	52	4.47

**Table 2:** Correlations of depressive symptoms among five measurement points and descriptive statistics.

	T1 ^a^	T2 ^a^	T3 ^a^	T4 ^a^	T5 ^a^
T1	1				
T2	0.476 **	1			
T3	0.401 **	0.613 **	1		
T4	0.448 **	0.582 **	0.628 **	1	
T5	0.384 **	0.538 **	0.611 **	0.736 **	1
Total ^b^	34.83 (8.91)	43.61 (9.09)	45.88 (9.31)	43.04 (10.01)	43.13 (10.45)
Girls ^b^	34.85 (8.56)	43.67 (9.08)	45.92 (9.23)	42.84 (9.89)	43.17 (10.16)
Boys ^b^	34.71 (10.32)	43.35 (9.14)	45.73 (9.65)	43.88 (10.50)	42.98 (11.65)

^**^p < 0.01. ^a^ T1–T5 different assessment time points. ^b^ means and standard deviation.

**Table 3:** Criteria used to decide on optimal solution for number of latent classes.

Classes	AIC	BIC	ABIC	Entropy	VLRT *p*	BLRT *p*
1	36,549.257	36,598.640	36,566.878			
2	36,506.360	36,580.434	36,532.793	0.838	0.0000	0.0000
3	36,480.465	36,579.230	36,515.708	0.768	0.0074	0.0000
4	36,452.717	36,576.174	36,496.771	0.618	0.0666	0.0074

## Data Availability

All data are available based on reasonable request.
